# Supplementary material for: Assessment of non-alcoholic fatty liver disease (NAFLD) severity with novel serum-based markers: A pilot study
Source: PLoS One. 2021 Nov 23;16(11):e0260313. doi: 10.1371/journal.pone.0260313 (PMC8610238; doi:10.1371/journal.pone.0260313)
Supplement: S1 Table — (DOCX) [file pone.0260313.s001.docx]

**S1 Table.** Geometric mean (95% CI) levels of serum-based markers, according to demographic factors.

|  | Age (years) | | BMI (kg/m^2^) | | Gender | |
| --- | --- | --- | --- | --- | --- | --- |
|  | **<50** | **≥50** | **<30** | **≥30** | **Male** | **Female** |
| n | 43 | 62 | 45 | 60 | 61 | 44 |
| Adiponectin (µg/ml) | 4.15  (3.63, 4.75) | 6.02  (5.17, 7.02) | 5.48  (4.40, 6.81) | 4.98  (4.42, 5.61) | 4.40  (3.88, 4.99) | 6.50  (5.42, 7.80) |
| Leptin (ng/ml) | 3.55  (2.56, 4.93) | 3.94  (2.90, 5.36) | 2.96  (2.17, 4.04) | 4.46  (3.28, 6.06) | 2.57  (1.94, 3.40) | 6.51  (4.82, 8.79) |
| Resistin (ng/ml) | 9.09  (7.41, 11.14) | 11.06  (9.70, 12.61) | 9.35  (7.71, 11.35) | 10.81  (9.39, 12.45) | 9.91  (8.54, 11.52) | 10.61  (8.84, 12.72) |
| TNFα (pg/ml) | 7.72  (6.98, 8.53) | 9.05  (8.16, 10.04) | 9.23  (8.01, 10.63) | 8.00  (7.39, 8.66) | 8.27  (7.68, 8.91) | 8.77  (7.56, 10.18) |
| IL-6 (pg/ml) | 1.18  (0.94, 1.48) | 1.92  (1.52, 2.41) | 1.34  (1.02, 1.77) | 1.74  (1.41, 2.15) | 1.56  (1.25, 1.96) | 1.57  (1.22, 2.03) |
| PAI-1 (ng/ml) | 28.98  (25.47, 32.98) | 23.25  (20.49, 26.38) | 25.43  (21.50, 30.07) | 25.50  (22.85, 28.47) | 25.33  (22.15, 28.95) | 25.68  (22.66, 29.10) |
| sIL-6 (ng/ml) | 1.17  (0.99, 1.40) | 1.37  (1.13, 1.67) | 1.20  (0.94, 1.52) | 1.35  (1.15, 1.58) | 1.18  (0.99, 1.40) | 1.46  (1.18, 1.80) |
| sTNFR1 (ng/ml) | 0.34  (0.31, 0.37) | 0.38  (0.34, 0.42) | 0.34  (0.30, 0.39) | 0.38  (0.34, 0.42) | 0.34  (0.31, 0.39) | 0.39  (0.34, 0.43) |
| sTNFR2 (ng/ml) | 0.23  (0.18, 0.30) | 0.33  (0.29, 0.39) | 0.28  (0.23, 0.34) | 0.29  (0.24, 0.35) | 0.27  (0.23, 0.32) | 0.31  (0.25, 0.39) |
| MMP9 (ng/ml) | 70.44  (57.64, 86.08) | 71.69  (62.34, 82.44) | 69.74  (56.13, 86.65) | 72.15  (63.40, 82.10) | 69.97  (60.08, 81.48) | 72.83  (60.80, 87.25) |
| Keratin-18 (U/L) | 230.3  (182.1, 291.3) | 191.5  (55.8, 235.4) | 165.8  (130.7, 210.4) | 239.0  (195.9, 291.4) | 248.6  (202.6, 305.2) | 161.5  (129.4, 201.5) |
| Ghrelin (ng/ml) | 13.14  (9.64, 17.9) | 15.94  (11.90, 21.37) | 12.90  (9.23, 18.01) | 16.22  (12.17, 21.44) | 15.37  (11.33, 20.85) | 13.88  (10.37, 18.58) |

BMI, Body Mass Index; TNFα, Tumour Necrosis Factor alpha; IL-6, Interleukin-6 ; PAI-1, Plasminogen Activator Inhibitor-1; sIL-6R, Interleukin-6 receptor; sTNFR1, soluble TNFα receptor 1; sTNFR2, soluble TNFα receptor 2; MMP-9, Matrix Metalloproteinase-9; µg, micrograms; ng, nanograms; pg, picograms; ml, millilitres.
