# Supplementary material for: Assessment of non-alcoholic fatty liver disease (NAFLD) severity with novel serum-based markers: A pilot study
Source: PLoS One. 2021 Nov 23;16(11):e0260313. doi: 10.1371/journal.pone.0260313 (PMC8610238; doi:10.1371/journal.pone.0260313)
Supplement: S2 Table — a. Geometric Mean (95% CI) levels of serum-based markers, according to Fibroscan result. p-values calculated using unpaired t-test (of log values)–no formal correction made for multiple testing. b. Geometric Mean (95% CI) levels of serum-based markers, according to CAP result. p-values calculated using unpaired t-test (of log values)–no formal correction made for multiple testing. (DOCX) [file pone.0260313.s002.docx]

**S2a Table. Geometric Mean (95% CI) levels of serum-based markers, according to Fibroscan result.** p-values calculated using unpaired t-test (of log values) – no formal correction made for multiple testing*.*

|  | Fibroscan result | |  |
| --- | --- | --- | --- |
|  | **<7.2 kPa** | **≥7.2 kPa** | **p-value** |
| Adiponectin (µg/ml) | 5.17 (4.46, 6.00) | 4.95 (4.17, 5.87) | 0.69 |
| Leptin (ng/ml) | 3.64 (2.74, 4.84) | 3.94 (2.64, 5.86) | 0.75 |
| Resistin (ng/ml) | 9.61 (8.14, 11.35) | 10.77 (9.19, 12.62) | 0.32 |
| TNFα (pg/ml) | **7.90 (7.31, 8.54)** | **9.58 (8.28, 11.08)** | **0.022** |
| IL-6 (pg/ml) | **1.25 (1.04, 1.51)** | **2.02 (1.50, 2.73)** | **0.007** |
| PAI-1 (ng/ml) | 25.81 (22.85, 29.15) | 25.62 (21.83, 30.06) | 0.9398 |
| sIL-6R (ng/ml) | 1.22 (1.01, 1.47) | 1.47 (1.20, 1.79) | 0.19 |
| sTNFR1 (ng/ml) | 0.35 (0.32, 0.39) | 0.37 (0.31, 0.43) | 0.68 |
| sTNFR2 (ng/ml) | 0.26 (0.21, 0.32) | 0.33 (0.27, 0.40) | 0.12 |
| MMP-9 (ng/ml) | **80.76 (69.99, 93.20)** | **57.91 (47.80, 70.16)** | **0.005** |
| Keratin 18 (U/L) | 194.2 (160.9, 234.4) | 225.9 (170.8, 298.6) | 0.35 |
| Ghrelin (ng/ml) | 12.27 (9.18, 16.40) | 20.69 (15.19, 28.19) | 0.018 |

TNFα, Tumour Necrosis Factor alpha; IL-6, Interleukin-6 ; PAI-1, Plasminogen Activator Inhibitor-1; sIL-6R, Interleukin-6 receptor; sTNFR1, soluble TNFα receptor 1; sTNFR2, soluble TNFα receptor 2; MMP-9, Matrix Metalloproteinase-9; kPa, kilopascal; U/L, Units per Litre; µg, micrograms; ng, nanograms; pg, picograms; ml, millilitres.

**S2b Table. Geometric Mean (95% CI) levels of serum-based markers, according to CAP result.** p-values calculated using unpaired t-test (of log values) – no formal correction made for multiple testing*.*

|  | CAP result | |  |
| --- | --- | --- | --- |
|  | **<302 dB/m** | **≥302dB/m** | **p-value** |
| Adiponectin (µg/ml) | 6.89 (4.41, 9.38) | 5.15 (4.28, 6.02) | 0.078 |
| Leptin (ng/ml) | 6.08 (0.18, 12.35) | 7.86 (5.24, 10.47) | 0.078 |
| Resistin (ng/ml) | 12.62 (8.31, 16.93) | 9.76 (8.04, 11.48) | 0.781 |
| TNFα (pg/ml) | 8.05 (6.11, 9.98) | 8.61 (7.70, 9.51) | 0.815 |
| IL-6 (pg/ml) | 1.71 (1.06, 2.36) | 2.21 (1.73, 2.68) | 0.069 |
| PAI-1 (ng/ml) | 24.92 (17.76, 32.09) | 27.87 (23.90, 31.85) | 0.344 |
| sIL-6R (ng/ml) | 1.53 (1.06, 2.01) | 1.42 (1.08, 1.77) | 0.858 |
| sTNFR1 (ng/ml) | 0.41 (0.31, 0.50) | 0.40 (0.34, 0.45) | 0.589 |
| sTNFR2 (ng/ml) | 0.41 (0.19, 0.63) | 0.33 (0.25, 0.43) | 0.706 |
| MMP-9 (ng/ml) | **91.23 (59.88, 122.58)** | **65.83 (54.37, 77.31)** | **0.002** |
| Keratin 18 (U/L) | **121.60 (82.95, 160.25)** | **335.10 (235.69, 434.52)** | **<0.001** |
| Ghrelin (ng/ml) | **13.43 (6.83, 20.03)** | **20.68 (15.64, 25.72)** | **0.012** |

TNFα, Tumour Necrosis Factor alpha; IL-6, Interleukin-6 ; PAI-1, Plasminogen Activator Inhibitor-1; sIL-6R, Interleukin-6 receptor; sTNFR1, soluble TNFα receptor 1; sTNFR2, soluble TNFα receptor 2; MMP-9, Matrix Metalloproteinase-9; kPa, kilopascal; U/L, Units per Litre; µg, micrograms; ng, nanograms; pg, picograms; ml, millilitres.
