# Supplementary material for: Assessment of non-alcoholic fatty liver disease (NAFLD) severity with novel serum-based markers: A pilot study
Source: PLoS One. 2021 Nov 23;16(11):e0260313. doi: 10.1371/journal.pone.0260313 (PMC8610238; doi:10.1371/journal.pone.0260313)
Supplement: S3 Table — a. Demographic factors associated with ghrelin concentrations in multivariable model. Results from linear regression model (log scale). Fit of model determined by examination of Pearson residual plots. b. Demographic factors associated with interleukin-6 (IL-6) concentrations in multivariable model. Results from linear regression model (log scale). Fit of model determined by examination of Pearson residual plots. c. Demographic factors associated with tumor necrosis factor a (TNFa) concentrations in multivariable model. Results from linear regression model (log scale). Fit of model determined by examination of Pearson residual plots. d. Demographic factors associated with metalloproteinase 9 (MMP9) concentrations in multivariable model. Results from linear regression model (log scale). Fit of model determined by examination of Pearson residual plots. (DOCX) [file pone.0260313.s003.docx]

**S3a Table. Demographic factors associated with ghrelin concentrations in multivariable model.** Results from linear regression model (log scale). Fit of model determined by examination of Pearson residual plots.

|  |  | Univariable | | | Multivariable | | |
| --- | --- | --- | --- | --- | --- | --- | --- |
|  |  | **Fold-increase** | **95% CI** | **p** | **Fold-increase** | **95% CI** | **p** |
| Sex | Male vs Female | 1.11 | 0.73, 1.69 | 0.6355 | 1.15 | 0.73, 1.80 | 0.5488 |
| Age (years) | <50 vs ≥50 | 0.82 | 0.54, 1.26 | 0.3688 | 0.88 | 0.55, 1.43 | 0.6190 |
| BMI (kg/m^2^) | <30 vs ≥30 | 0.80 | 0.52, 1.21 | 0.2841 | 0.85 | 0.55, 1.30 | 0.4475 |
| Diabetes | Yes vs No | 1.37 | 0.87, 2.15 | 0.1696 | 0.99 | 0.59, 1.66 | 0.9700 |
| Hyperlipidaemia | Yes vs No | 1.17 | 0.77, 1.77 | 0.4588 | 1.05 | 0.67, 1.64 | 0.8353 |
| Hypertension | Yes vs No | 1.65 | 1.10, 2.47 | 0.0155 | 1.57 | 0.98, 2.53 | 0.0643 |
| History of CVD | Yes vs No | 1.24 | 0.62, 2.50 | 0.5403 | 0.86 | 0.40, 1.86 | 0.7098 |

CI, confidence interval; BMI, Body Mass Index; kg, kilograms; m, metre; CVD, cardiovascular disease.

**S3b Table. Demographic factors associated with interleukin 6(IL-6) concentrations in multivariable model.** Results from linear regression model (log scale). Fit of model determined by examination of Pearson residual plots.

|  |  | Univariable | | | Multivariable | | |
| --- | --- | --- | --- | --- | --- | --- | --- |
|  |  | **Fold-increase** | **95% CI** | **p** | **Fold-increase** | **95% CI** | **p** |
| Sex | **Male vs Female** | 1.00 | 0.71, 1.39 | 0.9769 | 1.07 | 0.76, 1.50 | 0.6966 |
| Age (years) | **<50 vs ≥50** | 0.62 | 0.45, 0.85 | 0.0030 | **0.61** | 0.43, 0.88 | **0.0089** |
| BMI (kg/m^2^) | **<30 vs ≥30** | 0.77 | 0.55, 1.07 | 0.1244 | 0.74 | 0.54, 1.02 | 0.0710 |
| Diabetes | **Yes vs No** | 1.08 | 0.75, 1.55 | 0.6763 | 0.89 | 0.60, 1.32 | 0.5473 |
| Hyperlipidaemia | **Yes vs No** | 1.32 | 0.96, 1.83 | 0.0910 | 1.21 | 0.86, 1.70 | 0.2743 |
| Hypertension | **Yes vs No** | 1.19 | 0.86, 1.65 | 0.2968 | 0.97 | 0.67, 1.39 | 0.8665 |
| History of CVD | **Yes vs No** | 1.69 | 0.99, 2.89 | 0.0542 | 1.22 | 0.68, 2.16 | 0.5041 |

CI, confidence interval; BMI, Body Mass Index; kg, kilograms; m, metre; CVD, cardiovascular disease.

**S3c Table. Demographic factors associated with tumor necrosis factor a(TNFα) concentrations in multivariable model.** Results from linear regression model (log scale). Fit of model determined by examination of Pearson residual plots**.**

|  |  | | | Univariable | | | | | | Multivariable | | | | | |
| --- | --- | --- | --- | --- | --- | --- | --- | --- | --- | --- | --- | --- | --- | --- | --- |
|  |  | | | **Fold-increase** | | **95% CI** | | **p** | | **Fold-increase** | | **95% CI** | | **p** | |
| Sex | | **Male *vs* Female** | 0.94 | | 0.81, 1.09 | | 0.432 | | 1.02 | | 0.88, 1.18 | | 0.804 | |  |
| Age (years) | | **<50 *vs* ≥50** | 0.85 | | 0.74, 0.98 | | 0.029 | | 0.89 | | 0.76, 1.04 | | 0.149 | |  |
| BMI (kg/m^2^) | | **<30 *vs* ≥30** | 1.15 | | 1.00, 1.33 | | 0.054 | | 1.15 | | 1.00, 1.32 | | 0.059 | |  |
| Diabetes | | **Yes *vs* No** | 1.19 | | 1.02, 1.39 | | 0.028 | | 1.06 | | 0.89, 1.26 | | 0.488 | |  |
| Hyperlipidaemia | | **Yes *vs* No** | 1.15 | | 1.00, 1.33 | | 0.051 | | 1.09 | | 0.94, 1.27 | | 0.244 | |  |
| Hypertension | | **Yes *vs* No** | 1.17 | | 1.01, 1.35 | | 0.032 | | 1.15 | | 0.98, 1.35 | | 0.080 | |  |
| History of CVD | | **Yes *vs* No** | 0.90 | | 0.71, 1.14 | | 0.389 | | 0.78 | | 0.61, 1.00 | | 0.052 | |  |

CI, confidence interval; BMI, Body Mass Index; CVD, cardiovascular disease.

**S3d Table. Demographic factors associated with metalloproteinase 9 (MMP9) concentrations in multivariable model.** Results from linear regression model (log scale). Fit of model determined by examination of Pearson residual plots.

|  |  | Univariable | | | Multivariable | | |
| --- | --- | --- | --- | --- | --- | --- | --- |
|  |  | **Fold-increase** | **95% CI** | **p** | **Fold-increase** | **95% CI** | **p** |
| Sex | **Male vs Female** | 0.96 | 0.77, 1.20 | 0.729 | 0.91 | 0.71, 1.16 | 0.444 |
| Age (years) | **<50 *vs* ≥50** | 0.98 | 0.78, 1.23 | 0.879 | 1.05 | 0.81, 1.36 | 0.726 |
| BMI (kg/m^2^) | **<30 *vs* ≥30** | 0.97 | 0.77, 1.22 | 0.772 | 0.99 | 0.78, 1.26 | 0.943 |
| Diabetes | **Yes *vs* No** | 1.02 | 0.80, 1.31 | 0.863 | 0.96 | 0.72, 1.28 | 0.804 |
| Hyperlipidaemia | **Yes *vs* No** | 1.02 | 0.81, 1.28 | 0.876 | 1.00 | 0.79, 1.28 | 0.978 |
| Hypertension | **Yes *vs* No** | 1.00 | 0.80, 1.25 | 0.989 | 0.98 | 0.75, 1.27 | 0.869 |
| History of CVD | **Yes *vs* No** | 1.32 | 0.90, 1.94 | 0.162 | 1.41 | 0.92, 2.18 | 0.121 |

Abbreviations: CI, confidence interval; BMI, Body Mass Index; kg, kilograms; m, metre; CVD, cardiovascular disease.
