# Supplementary material for: Assessment of non-alcoholic fatty liver disease (NAFLD) severity with novel serum-based markers: A pilot study
Source: PLoS One. 2021 Nov 23;16(11):e0260313. doi: 10.1371/journal.pone.0260313 (PMC8610238; doi:10.1371/journal.pone.0260313)
Supplement: S4 Table — a. Factors associated with Fibroscan result ≥9.6 kPa. Logistic regression analysis in all study patients. b. Multi-variable analysis of factors associated with Fibroscan result ≥9.6 kPa in all study patients. (DOCX) [file pone.0260313.s004.docx]

**S4a Table:** Factors associated with Fibroscan result ≥9.6 kPa. Logistic regression analysis in all study patients.

|  |  | Univariable | | | Multivariable | | |
| --- | --- | --- | --- | --- | --- | --- | --- |
|  |  | **OR** | **95% CI** | **p** | **OR** | **95% CI** | **p** |
| Sex | Male vs Female | 0.59 | 0.21, 1.70 | 0.330 |  |  |  |
| Age (years) | ≥50 vs <50 | **0.06** | **0.01, 0.50** | **0.009** | **10.95** | **1.38, 89.64** | **0.026** |
| BMI (kg/m^2^) | ≥30 vs <30 | 1.27 | 0.44, 3.62 | 0.660 |  |  |  |
| Diabetes | Yes vs No | **4.83** | **1.59, 14.65** | **0.005** | 2.22 | 0.60, 8.16 | 0.230 |
| Hyperlipidaemia | Yes vs No | 1.07 | 0.38, 3.05 | 0.899 |  |  |  |
| Hypertension | Yes vs No | **4.63** | **1.39, 15.46** | **0.013** | 2.24 | 0.55, 9.16 | 0.261 |
| History of CVD | Yes vs No | 0.65 | 0.75, 5.67 | 0.698 |  |  |  |

OR, odds ratio; CI, confidence interval; BMI, Body Mass Index; CVD, cardiovascular disease.

**S4b Table.** Multi-variable analysis of factors associated with Fibroscan result ≥9.6 kPa in all study patients..

|  |  | Univariable | | | Multivariable | | |
| --- | --- | --- | --- | --- | --- | --- | --- |
|  |  | **OR** | **95% CI** | **p** | **OR** | **95% CI** | **p** |
| Sex | Male vs Female | 0.67 | 0.30, 1.49 | 0.323 |  |  |  |
| Age (years) | <50 vs ≥50 | **0.06** | **0.01, 0.50** | **0.009** | **0.098** | **0.01, 0.88** | **0.038** |
| BMI (kg/m^2^) | <30 vs ≥30 | 1.27 | 0.44, 3.62 | 0.660 |  |  |  |
| Diabetes | Yes vs No | **4.83** | **1.59, 14.65** | **0.005** | 1.85 | 0.42, 8.29 | 0.419 |
| Hyperlipidaemia | Yes vs No | 1.07 | 0.38, 3.05 | 0.899 |  |  |  |
| Hypertension | Yes vs No | **4.63** | **1.39, 15.46** | **0.013** | 1.73 | 0.36, 8.27 | 0.491 |
| History of CVD | Yes vs No | 0.65 | 0.75, 5.67 | 0.698 |  |  |  |
| Ghrelin | Per 1-log higher | **3.92** | **1.17. 13.13** | **0.027** | 2.48 | 0.58, 10.60 | 0.220 |
| TNFα | Per 1-log higher | **17.93** | **2.74, 155.01** | **0.001** | **56.51** | **1.97, 162.09** | **0.028** |
| IL-6 | Per 1-log higher | **8.31** | **1.26, 54.79** | **0.028** | 1.94 | 0.29, 2.84 | 0.493 |
| MMP-9 | Per 1-log higher | **0.35** | **0.04, 2.80** | **0.321** |  |  |  |

OR, odds ratio; CI, confidence interval; BMI, Body Mass Index; kg, kilograms; m, metre; CVD, cardiovascular disease; TNFα, Tumour Necrosis Factor alpha; IL-6, Interleukin-6; MMP-9, Matrix Metalloproteinase-9.
