# Supplementary material for: Assessment of non-alcoholic fatty liver disease (NAFLD) severity with novel serum-based markers: A pilot study
Source: PLoS One. 2021 Nov 23;16(11):e0260313. doi: 10.1371/journal.pone.0260313 (PMC8610238; doi:10.1371/journal.pone.0260313)
Supplement: S1 File — (DOCX) [file pone.0260313.s005.docx]

**Supplementary Data**

**Assessment of non-alcoholic fatty liver disease (NAFLD) severity with novel serum-based markers: A Pilot Study**

Atul Goyale^1^, Anjly Jain^1^, Colette Smith^2^, Margarita Papatheodoridi^3^, Marta Guerrero Misas^3^, Davide Roccarina^3^, Laura Iogna Prat^3^, Dimitri P Mikhailidis^1^, Devaki Nair^1*^, Emmanuel Tsochatzis^3 *^

**Contents**

**S1 Table……….. p. 2**

**S2a Table…… . p. 4**

**S2b Table………p. 5**

**S3a Table……… p. 6**

**S3b Table……… p. 7**

**S3c Table……… p. 8**

**S3d Table……… p. 9**

**S4a Table………. p. 10**

**S4b Table………. p.11**

**S1 Table.** Geometric mean (95% CI) levels of serum-based markers, according to demographic factors.

|  | Age (years) | | BMI (kg/m^2^) | | Gender | |
| --- | --- | --- | --- | --- | --- | --- |
|  | **<50** | **≥50** | **<30** | **≥30** | **Male** | **Female** |
| n | 43 | 62 | 45 | 60 | 61 | 44 |
| Adiponectin (µg/ml) | 4.15  (3.63, 4.75) | 6.02  (5.17, 7.02) | 5.48  (4.40, 6.81) | 4.98  (4.42, 5.61) | 4.40  (3.88, 4.99) | 6.50  (5.42, 7.80) |
| Leptin (ng/ml) | 3.55  (2.56, 4.93) | 3.94  (2.90, 5.36) | 2.96  (2.17, 4.04) | 4.46  (3.28, 6.06) | 2.57  (1.94, 3.40) | 6.51  (4.82, 8.79) |
| Resistin (ng/ml) | 9.09  (7.41, 11.14) | 11.06  (9.70, 12.61) | 9.35  (7.71, 11.35) | 10.81  (9.39, 12.45) | 9.91  (8.54, 11.52) | 10.61  (8.84, 12.72) |
| TNFα (pg/ml) | 7.72  (6.98, 8.53) | 9.05  (8.16, 10.04) | 9.23  (8.01, 10.63) | 8.00  (7.39, 8.66) | 8.27  (7.68, 8.91) | 8.77  (7.56, 10.18) |
| IL-6 (pg/ml) | 1.18  (0.94, 1.48) | 1.92  (1.52, 2.41) | 1.34  (1.02, 1.77) | 1.74  (1.41, 2.15) | 1.56  (1.25, 1.96) | 1.57  (1.22, 2.03) |
| PAI-1 (ng/ml) | 28.98  (25.47, 32.98) | 23.25  (20.49, 26.38) | 25.43  (21.50, 30.07) | 25.50  (22.85, 28.47) | 25.33  (22.15, 28.95) | 25.68  (22.66, 29.10) |
| sIL-6 (ng/ml) | 1.17  (0.99, 1.40) | 1.37  (1.13, 1.67) | 1.20  (0.94, 1.52) | 1.35  (1.15, 1.58) | 1.18  (0.99, 1.40) | 1.46  (1.18, 1.80) |
| sTNFR1 (ng/ml) | 0.34  (0.31, 0.37) | 0.38  (0.34, 0.42) | 0.34  (0.30, 0.39) | 0.38  (0.34, 0.42) | 0.34  (0.31, 0.39) | 0.39  (0.34, 0.43) |
| sTNFR2 (ng/ml) | 0.23  (0.18, 0.30) | 0.33  (0.29, 0.39) | 0.28  (0.23, 0.34) | 0.29  (0.24, 0.35) | 0.27  (0.23, 0.32) | 0.31  (0.25, 0.39) |
| MMP9 (ng/ml) | 70.44  (57.64, 86.08) | 71.69  (62.34, 82.44) | 69.74  (56.13, 86.65) | 72.15  (63.40, 82.10) | 69.97  (60.08, 81.48) | 72.83  (60.80, 87.25) |
| Keratin-18 (U/L) | 230.3  (182.1, 291.3) | 191.5  (55.8, 235.4) | 165.8  (130.7, 210.4) | 239.0  (195.9, 291.4) | 248.6  (202.6, 305.2) | 161.5  (129.4, 201.5) |
| Ghrelin (ng/ml) | 13.14  (9.64, 17.9) | 15.94  (11.90, 21.37) | 12.90  (9.23, 18.01) | 16.22  (12.17, 21.44) | 15.37  (11.33, 20.85) | 13.88  (10.37, 18.58) |

BMI, Body Mass Index; TNFα, Tumour Necrosis Factor alpha; IL-6, Interleukin-6 ; PAI-1, Plasminogen Activator Inhibitor-1; sIL-6R, Interleukin-6 receptor; sTNFR1, soluble TNFα receptor 1; sTNFR2, soluble TNFα receptor 2; MMP-9, Matrix Metalloproteinase-9; µg, micrograms; ng, nanograms; pg, picograms; ml, millilitres.

**S2a Table. Geometric Mean (95% CI) levels of serum-based markers, according to Fibroscan result.** p-values calculated using unpaired t-test (of log values) – no formal correction made for multiple testing*.*

|  | Fibroscan result | |  |
| --- | --- | --- | --- |
|  | **<7.2 kPa** | **≥7.2 kPa** | **p-value** |
| Adiponectin (µg/ml) | 5.17 (4.46, 6.00) | 4.95 (4.17, 5.87) | 0.69 |
| Leptin (ng/ml) | 3.64 (2.74, 4.84) | 3.94 (2.64, 5.86) | 0.75 |
| Resistin (ng/ml) | 9.61 (8.14, 11.35) | 10.77 (9.19, 12.62) | 0.32 |
| TNFα (pg/ml) | **7.90 (7.31, 8.54)** | **9.58 (8.28, 11.08)** | **0.022** |
| IL-6 (pg/ml) | **1.25 (1.04, 1.51)** | **2.02 (1.50, 2.73)** | **0.007** |
| PAI-1 (ng/ml) | 25.81 (22.85, 29.15) | 25.62 (21.83, 30.06) | 0.9398 |
| sIL-6R (ng/ml) | 1.22 (1.01, 1.47) | 1.47 (1.20, 1.79) | 0.19 |
| sTNFR1 (ng/ml) | 0.35 (0.32, 0.39) | 0.37 (0.31, 0.43) | 0.68 |
| sTNFR2 (ng/ml) | 0.26 (0.21, 0.32) | 0.33 (0.27, 0.40) | 0.12 |
| MMP-9 (ng/ml) | **80.76 (69.99, 93.20)** | **57.91 (47.80, 70.16)** | **0.005** |
| Keratin 18 (U/L) | 194.2 (160.9, 234.4) | 225.9 (170.8, 298.6) | 0.35 |
| Ghrelin (ng/ml) | 12.27 (9.18, 16.40) | 20.69 (15.19, 28.19) | 0.018 |

TNFα, Tumour Necrosis Factor alpha; IL-6, Interleukin-6 ; PAI-1, Plasminogen Activator Inhibitor-1; sIL-6R, Interleukin-6 receptor; sTNFR1, soluble TNFα receptor 1; sTNFR2, soluble TNFα receptor 2; MMP-9, Matrix Metalloproteinase-9; kPa, kilopascal; U/L, Units per Litre; µg, micrograms; ng, nanograms; pg, picograms; ml, millilitres.

**S2b Table. Geometric Mean (95% CI) levels of serum-based markers, according to CAP result.** p-values calculated using unpaired t-test (of log values) – no formal correction made for multiple testing*.*

|  | CAP result | |  |
| --- | --- | --- | --- |
|  | **<302 dB/m** | **≥302dB/m** | **p-value** |
| Adiponectin (µg/ml) | 6.89 (4.41, 9.38) | 5.15 (4.28, 6.02) | 0.078 |
| Leptin (ng/ml) | 6.08 (0.18, 12.35) | 7.86 (5.24, 10.47) | 0.078 |
| Resistin (ng/ml) | 12.62 (8.31, 16.93) | 9.76 (8.04, 11.48) | 0.781 |
| TNFα (pg/ml) | 8.05 (6.11, 9.98) | 8.61 (7.70, 9.51) | 0.815 |
| IL-6 (pg/ml) | 1.71 (1.06, 2.36) | 2.21 (1.73, 2.68) | 0.069 |
| PAI-1 (ng/ml) | 24.92 (17.76, 32.09) | 27.87 (23.90, 31.85) | 0.344 |
| sIL-6R (ng/ml) | 1.53 (1.06, 2.01) | 1.42 (1.08, 1.77) | 0.858 |
| sTNFR1 (ng/ml) | 0.41 (0.31, 0.50) | 0.40 (0.34, 0.45) | 0.589 |
| sTNFR2 (ng/ml) | 0.41 (0.19, 0.63) | 0.33 (0.25, 0.43) | 0.706 |
| MMP-9 (ng/ml) | **91.23 (59.88, 122.58)** | **65.83 (54.37, 77.31)** | **0.002** |
| Keratin 18 (U/L) | **121.60 (82.95, 160.25)** | **335.10 (235.69, 434.52)** | **<0.001** |
| Ghrelin (ng/ml) | **13.43 (6.83, 20.03)** | **20.68 (15.64, 25.72)** | **0.012** |

TNFα, Tumour Necrosis Factor alpha; IL-6, Interleukin-6 ; PAI-1, Plasminogen Activator Inhibitor-1; sIL-6R, Interleukin-6 receptor; sTNFR1, soluble TNFα receptor 1; sTNFR2, soluble TNFα receptor 2; MMP-9, Matrix Metalloproteinase-9; kPa, kilopascal; U/L, Units per Litre; µg, micrograms; ng, nanograms; pg, picograms; ml, millilitres.

**S3a Table. Demographic factors associated with ghrelin concentrations in multivariable model.** Results from linear regression model (log scale). Fit of model determined by examination of Pearson residual plots.

|  |  | Univariable | | | Multivariable | | |
| --- | --- | --- | --- | --- | --- | --- | --- |
|  |  | **Fold-increase** | **95% CI** | **p** | **Fold-increase** | **95% CI** | **p** |
| Sex | Male vs Female | 1.11 | 0.73, 1.69 | 0.6355 | 1.15 | 0.73, 1.80 | 0.5488 |
| Age (years) | <50 vs ≥50 | 0.82 | 0.54, 1.26 | 0.3688 | 0.88 | 0.55, 1.43 | 0.6190 |
| BMI (kg/m^2^) | <30 vs ≥30 | 0.80 | 0.52, 1.21 | 0.2841 | 0.85 | 0.55, 1.30 | 0.4475 |
| Diabetes | Yes vs No | 1.37 | 0.87, 2.15 | 0.1696 | 0.99 | 0.59, 1.66 | 0.9700 |
| Hyperlipidaemia | Yes vs No | 1.17 | 0.77, 1.77 | 0.4588 | 1.05 | 0.67, 1.64 | 0.8353 |
| Hypertension | Yes vs No | 1.65 | 1.10, 2.47 | 0.0155 | 1.57 | 0.98, 2.53 | 0.0643 |
| History of CVD | Yes vs No | 1.24 | 0.62, 2.50 | 0.5403 | 0.86 | 0.40, 1.86 | 0.7098 |

CI, confidence interval; BMI, Body Mass Index; kg, kilograms; m, metre; CVD, cardiovascular disease.

**S3b Table. Demographic factors associated with interleukin 6(IL-6) concentrations in multivariable model.** Results from linear regression model (log scale). Fit of model determined by examination of Pearson residual plots.

|  |  | Univariable | | | Multivariable | | |
| --- | --- | --- | --- | --- | --- | --- | --- |
|  |  | **Fold-increase** | **95% CI** | **p** | **Fold-increase** | **95% CI** | **p** |
| Sex | **Male vs Female** | 1.00 | 0.71, 1.39 | 0.9769 | 1.07 | 0.76, 1.50 | 0.6966 |
| Age (years) | **<50 vs ≥50** | 0.62 | 0.45, 0.85 | 0.0030 | **0.61** | 0.43, 0.88 | **0.0089** |
| BMI (kg/m^2^) | **<30 vs ≥30** | 0.77 | 0.55, 1.07 | 0.1244 | 0.74 | 0.54, 1.02 | 0.0710 |
| Diabetes | **Yes vs No** | 1.08 | 0.75, 1.55 | 0.6763 | 0.89 | 0.60, 1.32 | 0.5473 |
| Hyperlipidaemia | **Yes vs No** | 1.32 | 0.96, 1.83 | 0.0910 | 1.21 | 0.86, 1.70 | 0.2743 |
| Hypertension | **Yes vs No** | 1.19 | 0.86, 1.65 | 0.2968 | 0.97 | 0.67, 1.39 | 0.8665 |
| History of CVD | **Yes vs No** | 1.69 | 0.99, 2.89 | 0.0542 | 1.22 | 0.68, 2.16 | 0.5041 |

CI, confidence interval; BMI, Body Mass Index; kg, kilograms; m, metre; CVD, cardiovascular disease.

**S3c Table. Demographic factors associated with tumor necrosis factor a(TNFα) concentrations in multivariable model.** Results from linear regression model (log scale). Fit of model determined by examination of Pearson residual plots**.**

|  |  | | | Univariable | | | | | | Multivariable | | | | | |
| --- | --- | --- | --- | --- | --- | --- | --- | --- | --- | --- | --- | --- | --- | --- | --- |
|  |  | | | **Fold-increase** | | **95% CI** | | **p** | | **Fold-increase** | | **95% CI** | | **p** | |
| Sex | | **Male *vs* Female** | 0.94 | | 0.81, 1.09 | | 0.432 | | 1.02 | | 0.88, 1.18 | | 0.804 | |  |
| Age (years) | | **<50 *vs* ≥50** | 0.85 | | 0.74, 0.98 | | 0.029 | | 0.89 | | 0.76, 1.04 | | 0.149 | |  |
| BMI (kg/m^2^) | | **<30 *vs* ≥30** | 1.15 | | 1.00, 1.33 | | 0.054 | | 1.15 | | 1.00, 1.32 | | 0.059 | |  |
| Diabetes | | **Yes *vs* No** | 1.19 | | 1.02, 1.39 | | 0.028 | | 1.06 | | 0.89, 1.26 | | 0.488 | |  |
| Hyperlipidaemia | | **Yes *vs* No** | 1.15 | | 1.00, 1.33 | | 0.051 | | 1.09 | | 0.94, 1.27 | | 0.244 | |  |
| Hypertension | | **Yes *vs* No** | 1.17 | | 1.01, 1.35 | | 0.032 | | 1.15 | | 0.98, 1.35 | | 0.080 | |  |
| History of CVD | | **Yes *vs* No** | 0.90 | | 0.71, 1.14 | | 0.389 | | 0.78 | | 0.61, 1.00 | | 0.052 | |  |

CI, confidence interval; BMI, Body Mass Index; CVD, cardiovascular disease.

**S3d Table. Demographic factors associated with metalloproteinase 9 (MMP9) concentrations in multivariable model.** Results from linear regression model (log scale). Fit of model determined by examination of Pearson residual plots.

|  |  | Univariable | | | Multivariable | | |
| --- | --- | --- | --- | --- | --- | --- | --- |
|  |  | **Fold-increase** | **95% CI** | **p** | **Fold-increase** | **95% CI** | **p** |
| Sex | **Male vs Female** | 0.96 | 0.77, 1.20 | 0.729 | 0.91 | 0.71, 1.16 | 0.444 |
| Age (years) | **<50 *vs* ≥50** | 0.98 | 0.78, 1.23 | 0.879 | 1.05 | 0.81, 1.36 | 0.726 |
| BMI (kg/m^2^) | **<30 *vs* ≥30** | 0.97 | 0.77, 1.22 | 0.772 | 0.99 | 0.78, 1.26 | 0.943 |
| Diabetes | **Yes *vs* No** | 1.02 | 0.80, 1.31 | 0.863 | 0.96 | 0.72, 1.28 | 0.804 |
| Hyperlipidaemia | **Yes *vs* No** | 1.02 | 0.81, 1.28 | 0.876 | 1.00 | 0.79, 1.28 | 0.978 |
| Hypertension | **Yes *vs* No** | 1.00 | 0.80, 1.25 | 0.989 | 0.98 | 0.75, 1.27 | 0.869 |
| History of CVD | **Yes *vs* No** | 1.32 | 0.90, 1.94 | 0.162 | 1.41 | 0.92, 2.18 | 0.121 |

Abbreviations: CI, confidence interval; BMI, Body Mass Index; kg, kilograms; m, metre; CVD, cardiovascular disease.

**Table S4a:** Factors associated with Fibroscan result ≥9.6 kPa. Logistic regression analysis in all study patients.

|  |  | Univariable | | | Multivariable | | |
| --- | --- | --- | --- | --- | --- | --- | --- |
|  |  | **OR** | **95% CI** | **p** | **OR** | **95% CI** | **p** |
| Sex | Male vs Female | 0.59 | 0.21, 1.70 | 0.330 |  |  |  |
| Age (years) | ≥50 vs <50 | **0.06** | **0.01, 0.50** | **0.009** | **10.95** | **1.38, 89.64** | **0.026** |
| BMI (kg/m^2^) | ≥30 vs <30 | 1.27 | 0.44, 3.62 | 0.660 |  |  |  |
| Diabetes | Yes vs No | **4.83** | **1.59, 14.65** | **0.005** | 2.22 | 0.60, 8.16 | 0.230 |
| Hyperlipidaemia | Yes vs No | 1.07 | 0.38, 3.05 | 0.899 |  |  |  |
| Hypertension | Yes vs No | **4.63** | **1.39, 15.46** | **0.013** | 2.24 | 0.55, 9.16 | 0.261 |
| History of CVD | Yes vs No | 0.65 | 0.75, 5.67 | 0.698 |  |  |  |

OR, odds ratio; CI, confidence interval; BMI, Body Mass Index; CVD, cardiovascular disease.

**Table S4b.** Multi-variable analysis of factors associated with Fibroscan result ≥9.6 kPa in all study patients..

|  |  | Univariable | | | Multivariable | | |
| --- | --- | --- | --- | --- | --- | --- | --- |
|  |  | **OR** | **95% CI** | **p** | **OR** | **95% CI** | **p** |
| Sex | Male vs Female | 0.67 | 0.30, 1.49 | 0.323 |  |  |  |
| Age (years) | <50 vs ≥50 | **0.06** | **0.01, 0.50** | **0.009** | **0.098** | **0.01, 0.88** | **0.038** |
| BMI (kg/m^2^) | <30 vs ≥30 | 1.27 | 0.44, 3.62 | 0.660 |  |  |  |
| Diabetes | Yes vs No | **4.83** | **1.59, 14.65** | **0.005** | 1.85 | 0.42, 8.29 | 0.419 |
| Hyperlipidaemia | Yes vs No | 1.07 | 0.38, 3.05 | 0.899 |  |  |  |
| Hypertension | Yes vs No | **4.63** | **1.39, 15.46** | **0.013** | 1.73 | 0.36, 8.27 | 0.491 |
| History of CVD | Yes vs No | 0.65 | 0.75, 5.67 | 0.698 |  |  |  |
| Ghrelin | Per 1-log higher | **3.92** | **1.17. 13.13** | **0.027** | 2.48 | 0.58, 10.60 | 0.220 |
| TNFα | Per 1-log higher | **17.93** | **2.74, 155.01** | **0.001** | **56.51** | **1.97, 162.09** | **0.028** |
| IL-6 | Per 1-log higher | **8.31** | **1.26, 54.79** | **0.028** | 1.94 | 0.29, 2.84 | 0.493 |
| MMP-9 | Per 1-log higher | **0.35** | **0.04, 2.80** | **0.321** |  |  |  |

OR, odds ratio; CI, confidence interval; BMI, Body Mass Index; kg, kilograms; m, metre; CVD, cardiovascular disease; TNFα, Tumour Necrosis Factor alpha; IL-6, Interleukin-6; MMP-9, Matrix Metalloproteinase-9.
