# Supplementary material for: Assessment of non-alcoholic fatty liver disease (NAFLD) severity with novel serum-based markers: A pilot study
Source: PLoS One. 2021 Nov 23;16(11):e0260313. doi: 10.1371/journal.pone.0260313 (PMC8610238; doi:10.1371/journal.pone.0260313)
Supplement: Attachment — Submitted filename: Response to reviewers.docx [file pone.0260313.s008.docx]

**RESPONSE TO REVIEWERS**

**Manuscript Ref. PONE-D-21-17417**

Dear Dr Strnad,

We would like to thank you for your consideration of our manuscript entitled “**Assessment of non-alcoholic fatty liver disease (NAFLD) severity with novel serum based markers: A Pilot Study**” with minor revisions.

We have tried to address all comments from the reviewers and we are looking forward to your final decision regarding the publication of our study.

Below you will find, in blue, a point-by-point response to the reviewers’ comments and concerns. All page numbers refer to the revised manuscript files.

In our revised main manuscript and supplementary material, you will find all the changes/new additions in red font.

**Journal Requirements**

Please review your reference list to ensure that it is complete and correct. If you have cited papers that have been retracted, please include the rationale for doing so in the manuscript text, or remove these references and replace them with relevant current references. Any changes to the reference list should be mentioned in the rebuttal letter that accompanies your revised manuscript. If you need to cite a retracted article, indicate the article’s retracted status in the References list and also include a citation and full reference for the retraction notice.

We have reviewed our reference list carefully and we have not found any retracted article.

We have now revised the order of the citations, following the addition of 2 more references, according to Reviewer’s comment no 2, as you will see in the revised manuscript.

**Reviewers’ Comments to the Author**

**Reviewer #1:** The authors addressed the majority of the raised comments in their revision and performed the suggested additional analyses. The manuscript has improved by this revision.

**Reviewer #2:** The comments of the reviewers have been mainly addressed; however, some minor corrections are still required:

1. Discussion page 22: "a recent systemic review...." replace reference 41 with reference 42.

Thank you for your comment. We have now corrected this to cite the correct study, which is no 44 in the updated reference list, after the additions mentioned in the next comment.

2. Discussion page 22: "This is contrary to previous studies and a recent systemic review exploring the role keratin-18 in NAFLD". This sentence should be corrected since the mentioned study (not studies) or review article did not evaluate or discuss an association of keratin-18 with fibroscan values but of keratin-18 fragments with histological fibrosis. It would be better to write “the role of keratin-18 for fibrosis in NAFLD”. Further studies indicating a correlation of keratin-18 fragments with fibrosis progression in NAFLD might be considered (Tamimi T et al., J Hepatol 2011; 54:1224-29; Diab DL et al., Clinical Gastroenterol Hepatol 2008; 6(11):1249-54).

Thank you for this comment. We have now updated this part of our discussion and we have added the suggested references as requested, (ref no 42, 43).

3. S1 and S2aTables: Replace Cytokeratin-18 with Keratin-18.

Thank you for this comment. We have now amended the typo in the updated Suppl. Material.
